# Comparative genomics of lactic acid bacteria reveals a niche-specific gene set

**DOI:** 10.1186/1471-2180-9-50

**Published:** 2009-03-05

**Authors:** Orla O'Sullivan, John O'Callaghan, Amaia Sangrador-Vegas, Olivia McAuliffe, Lydia Slattery, Pawel Kaleta, Michael Callanan, Gerald F Fitzgerald, R Paul Ross, Tom Beresford

**Affiliations:** 1 Teagasc, Moorepark Food Research Centre, Moorepark, Fermoy, Co. Cork, Ireland; 2 Alimentary Pharmabiotic Centre, Cork, Ireland; 3 Department of Microbiology, University College Cork, Ireland

## Abstract

**Background:**

The recently sequenced genome of *Lactobacillus helveticus *DPC4571 [1] revealed a dairy organism with significant homology (75% of genes are homologous) to a probiotic bacteria *Lb. acidophilus *NCFM [2]. This led us to hypothesise that a group of genes could be determined which could define an organism's niche.

**Results:**

Taking 11 fully sequenced lactic acid bacteria (LAB) as our target, (3 dairy LAB, 5 gut LAB and 3 multi-niche LAB), we demonstrated that the presence or absence of certain genes involved in sugar metabolism, the proteolytic system, and restriction modification enzymes were pivotal in suggesting the niche of a strain. We identified 9 niche specific genes, of which 6 are dairy specific and 3 are gut specific. The dairy specific genes identified in *Lactobacillus helveticus *DPC4571 were lhv_1161 and lhv_1171, encoding components of the proteolytic system, lhv_1031 lhv_1152, lhv_1978 and lhv_0028 encoding restriction endonuclease genes, while bile salt hydrolase genes lba_0892 and lba_1078, and the sugar metabolism gene lba_1689 from *Lb. acidophilus *NCFM were identified as gut specific genes.

**Conclusion:**

Comparative analysis revealed that if an organism had homologs to the dairy specific geneset, it probably came from a dairy environment, whilst if it had homologs to gut specific genes, it was highly likely to be of intestinal origin.

We propose that this "barcode" of 9 genes will be a useful initial guide to researchers in the LAB field to indicate an organism's ability to occupy a specific niche.

## Background

The LAB represents a group of organisms that are functionally related by their general ability to produce lactic acid during homo- or hetro-fermentative metabolism. They are predominantly Gram-positive, non-sporulating facultative anaerobic bacteria and have been isolated from sources as diverse as plants, animals and humans (for recent reviews on LAB see [3-7]). LAB can be sub-classified into 7 phylogenetic clades:*Lactococcus, Lactobacillus, Enterococcus, Pediococcus, Streptococcus, Leuconostoc *and *Oenococcus *[8]. They represent the single most exploited group of bacteria in the food industry, playing crucial roles in the fermentation of dairy products, meat and vegetables, as well as in the production of wine, coffee, cocoa and sourdough. This is reflected in the fact that to date (July 2008), 65 LAB genomes are either completely sequenced or in progress (source http://www.ncbi.nlm.nih.gov). Some LAB, such as *Lb. rhamnosus *ATCC 53013 and *Lb. acidophilus *NCFM have been shown to be probiotic, which is defined by the World Health Organisation as: *'Live microorganisms which when administered in adequate amounts confer a health benefit on the host'*. [9] LAB are also a reservoir for antimicrobial peptides, such as bacteriocins. There are numerous examples of bacteriocin producing LAB -one of the most recent being *Lb. salivarius *UCC118, which was shown to be effective in reducing *L. monocytogenes *infections in mice [10]. However, members of the LAB can also be important pathogens, e.g. several *Streptococcus *and *Enterococcus *species. Such species are commonly found in the human and animal GI tract and can occasionally cause disease. Diseases caused by colonisation of pathogenic LAB include urinary tract infections, bacteremia, bacterial endocarditis, diverticulitis, and meningitis.

Members of the LAB group have close phylogenetic relationships largely due to their sharing relatively small, AT-rich genomes (~2.4 Mb) and common metabolic pathways [8]. Despite their phylogenetic closeness, the LAB occupy a diverse set of ecological niches including fermenting plants, milk, wine, sour-dough, the human and animal GI tract and the oral cavities of vertebrates. Such niche diversity among closely-related species suggests considerable genetic adaptation during their evolution.

The recently sequenced dairy culture *Lb. helveticus *DPC4571 [1], has 98.4% 16s ribosomal RNA identity to the gut organism *Lb. acidophilus *NCFM [2]. This gave us a unique opportunity to investigate two very similar organisms occupying extremely different niches and led us to investigate if we could define a specific gene set which is associated with niche adaptation in LAB. Phylogenetically, both *Lb. helveticus *and *Lb. acidophilus *branch together with other gut bacteria. In recent years, numerous comparative studies of LAB [11-16] have made apparent that comparative genomics analysis will quickly reveal both the conserved and unique components of LAB that occupy different environmental niches. Knowledge of key gene sets that could promote a gut or dairy lifestyle could be very useful in guiding strain selection for multiple roles, either as probiotic or bioprocessing/fermentation cultures.

Our objective in this study was to take the differences in the phylogenetically related species; *Lb. helveticus *and *Lb. acidophilus *and investigate if we could define a niche specific gene-set, or a "barcode", which would help inform on the origin of particular strains of LAB.

## Results and discussion

**Figure 1:**
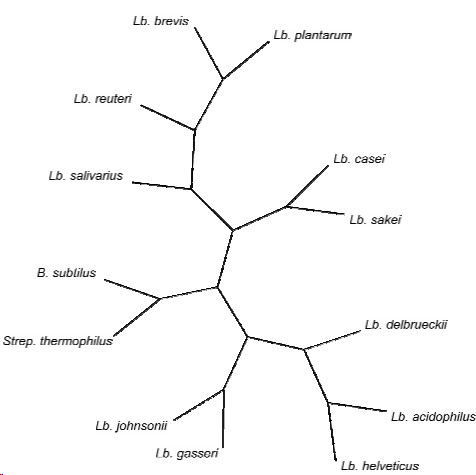
**Phylogenetic supertree of the eleven selected lactic acid bacteria and *B. subtilus***. The supertree was calculated form 47 individual ribosomal protein trees. All branches are supported at > 75% bootstrap values.

Although *Lb. helveticus *DPC4571 and *Lb. acidophilus *NCFM share remarkable genomic homology (16S rRNA sequence shares 98.4% identity) and conserved gene synteny, they occupy distinctly different niches (*Lb. helveticus *DPC4571 is a dairy organism while *Lb. acidophilus *NCFM is a gut organism). Analysis of the completed genome sequences revealed that 75% of predicted DPC4571 ORFs have orthologues in the *Lb. acidophilus *NCFM genome (orthology being defined as BLASTP *E *value < 10^-20^). We confirmed the positioning of *Lb. helveticus *DPC4571 by constructing a phylogenetic tree with concatenated alignments of 47 ribosomal proteins (Fig. 1), an approach shown to improve the resolution and robustness of phylogenetic analyses [17].

Focusing on the differences between the two genomes, DPC4571 has 123 (non-IS element) genes which are not found in NCFM while the NCFM strain has 503 genes not found in DPC4571. This gave us a starting point of 626 potential niche-specific genes, with the "DPC4571 only" genes being potential dairy-specific genes and the "NCFM only" genes being potential gut-specific genes. Of the 503 "NCFM only" genes, analysis of sequence data identified a number of IS element-associated gene losses from *Lb. helveticus *DPC4571, including ten interrupted genes and predicted deletions at 31 separate loci. These deletions were located in a number of genes whose loss would be expected to affect functionality in either a dairy and a non-dairy environment [1]. Interestingly, many of the genetic complement that distinguishes DPC4571 from NCFM appeared to be dairy- or gut-specific from a functional perspective. Survival and colonisation of the human gut relies on the presence of certain genes [18], such as those involved in (complex) sugar metabolism, and bile salt hydrolysis [4,18,19]. On the other hand, in order to survive in a dairy environment organisms appear to conserve specific genes involved in fatty acid degradation and proteolysis [3,4].

To this end, we investigated whether any of the candidate genes, 123 DPC4571 "dairy" genes or the 503 NCFM "gut" genes, could be used to identify a micro-organisms niche. An unbiased homology search with each of the candidate genes was executed against our initial selection of 11 genomes (table 1). These 11 genomes were selected on the basis that they were phylogenetically related to *Lb. helveticus *DPC4571 and *Lb. acidophilus *NCFM, they were fully sequenced genomes and they were isolated from either a dairy or gut environment or were capable of surviving in both. A gene was deemed a gut identifier gene if it has a homologue present in the 4 gut genomes and absent from the 3 dairy genomes. Conversely, a gene was deemed a dairy identifier if it had a homologue in the 3 dairy organisms but absent from the gut organisms. Criteria for homologue detection were a threshold of 1e^-10 ^and greater than 30% identity. Therefore, an organism could potentially survive a dairy environment if it contains dairy genes and an organism could potentially survive the gut if it contains gut genes. Based on these criteria, we identified 9 genes (table 2) that appear to be niche-specific. Simultaneously to this unbiased homology search we identified phenotypic groups of what we deemed to be desirable niche characteristics, namely genes involved in fatty acid metabolism, proteolysis and restriction modification systems, for the dairy environment [3,4] and for the gut environment genes involved in sugar metabolism, cell- wall and mucus binding and sugar metabolism [4,18,19]. Using literature searches and analysis using the ERGO database we identified the genes involved in these groupings and a blast search was performed with all genes within the groups against the same 11 genome group using the same selection criteria. Interestingly the unbiased and biased methods of identifying the barcode yielded the same 9-gene set. Furthermore, those organisms which can survive in multiple niches, namely *Lb. sakei *subsp.*sakei *23 K *Lb. brevis *ATCC367 and *Lb. plantarum *WCFS1 contained both dairy-specific and gut-specific genes. Multi-niche organisms will contain some genes from both the dairy and gut gene-set. To validate these niche-specific genes, we performed a broader BLAST search on a non-redundant database, containing all genes submitted to the NCBI database, from both fully and partially sequenced genomes, to ensure that the genes did not occur in any other dairy or gut organisms outside our selection. As with the unbiased and biased tests criteria for homologue detection were a threshold of 1e^-10 ^and greater than 30% identity. Particularly, the niche-specific genes could be categorised into four general functional classes i.e. sugar metabolism, the proteolytic system, restriction modification systems and bile salt hydrolysis. A detailed description of the LAB barcode genes will now be discussed.

**Table 1:** General genome features of eleven completely sequenced LAB.

Genome Features	*Lb. helveticus *DPC4571	*Lb. acidophilus *NCFM	*Lb. Johnsonii *NCC533	*Lb. sakei *23K	*Lb. salivarius *UCC118	*Lb. delbrueckii *subsp.*bulgaricus *ATCC11842	*Lb. plantarum *WCFS1	*S. thermophilus *LMG18311	*Lb. brevis *ATCC3567	*Lb. reuteri F25*	*Lb. gasseri ATCC 33323*
Length (bp)	2080931	1993564	1922676	1884664	1827111	1864998	3308274	1796846	2291220	2039414	1894360
G+C content (%)	37.8	34.7	34.6	41.3	32.9	49.0	44.4	39.0	46.0	38.0	35.0
Gene number	1618	1864	1821	1884	1765	1562	3051	1890	2314	1820	1898
Pseudogenes	217	0	0	30	49	533	39	180	49	0	48

**Table 2:** Niche Specific Genes

Dairy Specific Genes	Gut Specific Genes
**1) Proteolytic System**	**1) Bile Salt Hydrolysis**
Carboxypeptidase (lhv_1161, lhv_1171)	Bile Salt Hydrolase (lba_0892, lba_1078)
**2) R/M system**	**2) Sugar metabolism**
Restriction Modification enzyme Type I (lhv_1031, lhv_1152, lhv_1978) Restriction Modification Enzyme Type III (lhv_0028)	Maltose-6-phosphate glucosidase (lba_1689)

### Sugar Metabolism

Maltose-6-phosphate glycosidase (lba_1689 in *Lb. acidophilus *NCFM) is found solely in gut organisms and is absent even in multi-niche organism. Further analysis of this gene by BLAST comparison to all of the LAB genomes sequenced indicated that similar proteins are only present in *Lb. acidophilus, Lb. johnsonii*, *Lb. casei, Enterococcus faecalis*, *E. faecium *and *Streptococcus suis*. The three lactobacilli listed are classified as commensal gut strains, while the enterococci and *S. suis *are also considered commensal gut bacteria, associated more with humans and animals than with the dairy environment. Maltose uptake and metabolism in LAB can occur by 4 different mechanisms, as discussed by Le Breton *et al*. 2005 [20]. In two of these, maltose is taken into the cytoplasm by a permease; it is not phosphorylated and therefore, maltose-6-phosphate glycosidase is not required. In the other systems described, a phosphotransferase (PTS) is used to transport maltose and therefore, there is no necessity to assimilate the resulting maltose-6-phosphate. Metabolism of maltose-6-phosphate either occurs by a maltose-6-phosphate phosphorylase, converting maltose to glucose-1-phosphate and glucose-6-phosphate, or a maltose-6-phosphate glycosidase, converting maltose to glucose and glucose-6-phosphate. It is the latter mechanism that appears to be present in the 'gut' strains.

An analysis of 40 strains of LAB demonstrated that 32 of the strains could metabolise maltose and of these, 20 used a permease to transport maltose into the cell followed by conversion to glucose and β-glucose-1-phosphate by maltose phosphorylase [21]. The PTS/maltose-6-phosphate glycosidase pathway is therefore less common than the alternative mechanisms. Maltose is one of the least abundant disaccharides in the environment. It is present in germinating grain due to the action of amylases on starch and also presumably in other locations where starch breakdown products are present, such as in the gut. There is a possible bioenergetic advantage to cells with a PTS system; if phosphorylation is coupled to transport, it saves the energy required to phosphorylate within the cytoplasm, an important attribute if maltose were a significant energy source. It is notable that the PTS/glycosidase systems seem to be present in gut/commensal bacteria and others such as *Clostridium difficile *that can colonise the gut. Therefore, it would appear that adaptation to the intestinal niche seems to be associated with the presence of substantially higher numbers of genes encoding glycosidase enzymes, particularly those involved in the hydrolysis of disaccharides and oligosaccharides of plant origin. Genes for the metabolism of sugars other than lactose are almost entirely absent from the more nutritionally fastidious dairy strains.

Another interesting observation was that the degree of similarity between the genes/protein sequences from *Lb. helveticus *DPC4571 and *Lb. acidophilus *NCFM was generally much higher than between *Lb. acidophilus *NCFM and any of the other strains. While *Lb. acidophilus *NCFM and the other gut and multi-environment strains had very similar complements of glycosidase genes, the sequence similarity was much lower (with the exception of a few *Lb. johnsonii *genes) than between the NCFM/DPC4571 sequences, even though there were substantial differences in glycosidase gene content between *Lb. acidophilus *NCFM and *Lb. helveticus *DPC4571. The loss of a significant number of glycosidase genes together with the high degree of similarity between the remaining genes suggests that *Lb. helveticus *DPC4571 has undergone a relatively recent loss of sugar metabolism capacity relative to its divergence from *Lb. acidophilus *NCFM.

Of the sugar metabolism genes analysed, only one (lba_1689) can be used in our barcode as a gut organism indicator.

### Bile Salt Hydrolases

Intestinal bacteria can experience a wide number of stresses in the intestinal tract including those caused by low pH and presence of bile. In this respect, bile salt tolerance is thought to be an important aspect of survival for bacteria which inhabit the intestinal tract. Most intestinal isolates of lactobacilli and some lactobacilli involved in food fermentations exhibit bile salt hydrolase activity [22,23]. These enzymes catalyze the hydrolysis of conjugated bile acids, which enter the small bowel in bile and are important for the emulsification, digestion and absorption of dietary lipids present in the proximal small bowel [24]. It has been suggested that deconjugation of bile acids is a detoxification method and protects the cells from conjugated bile. Conversely, negative effects of bile salt hydrolase activity have also been reported including cases of contaminated small bowel syndrome, impaired lipid absorption, gallstone formation, and increased risk of colon cancer [25].

In *Lactobacillus*-free mice, bile salt hydrolase activity was reduced by 87%, revealing that lactobacilli are the main contributors to bile salt hydrolysis [23]. The presence of bile salts is an inherent stress associated with the gut environment and loss of bile salt hydrolase (*bsh*) genes is likely to result from adaptation to non-gut environments where bile salts are absent [26]. While it is indeed possible for *Lb. johnsonii *to persist in the mouse gut with all three of its *bsh *genes inactivated [27], the loss of a single physiological function does not necessarily mean that an organism changes its niche suitability. We would contend that while bile salt hydrolase genes are not essential for gut persistence the likelihood is that their presence increases the fitness of strains that possess them to exist in the gut environment and that it is extremely likely that gut strains will contain functional *bsh *genes. Accordingly, it would be expected that the *bsh *genes would only be present in the gut and multi-niche bacteria [28]. There are two *bsh *genes in *Lb. acidophilus *NCFM *bshA *(lba_0892) and *bshB *(lba_1078) [14], both of which were only found in the other gut associated organisms. More notably, on closer inspection we discovered that a *bsh *gene is present in *Lb. helveticus *DPC4571 but it has a frame-shift mutated which renders it non-functional. This suggests a common ancestry between *Lb. acidophilus *and *Lb. helveticus *and a recent loss of function in *Lb. helveticus*. Upon performing a wider BLAST search, it was discovered that both the *bshA *and *bshB *genes only occurred in organisms capable of gut survival, including *E. faecium, Clostridium perfringens, Listeria monocytogenes, Ruminococcus obeumand *and *Bifidobacterium bifidum*, thus making the genes *Lb. acidophilus *NCFM *bshA *(lba_8920) and *bshB *(lba_1078) ideal candidates for our barcode to identify gut organisms.

### The Proteolytic System

The proteolytic system of lactobacilli and other LAB, organisms which are fastidious in their amino acid requirements, is of importance from a dairy perspective in that it allows survival in milk and other dairy environments where the natural free amino acid concentrations are very low [29]. The combined action of proteinases and peptidases generates essential amino acids and small peptides during growth in the dairy environment. The system is also of major industrial importance due to its contribution to the development of the organoleptic properties of fermented milk products[30]. In cheese manufacturing, cell envelope proteinases (CEPs) play a pivotal role in the production of flavour compounds. Characterised peptidases such as PepN, PepX, PePO2 and PEPO3 are involved in the breakdown of hydrophobic peptides which could otherwise lead to bitterness in cheese. Combining LAB with different peptidase activity has been shown to reduce such bitterness [31]*L. lactis *and *Lb. helveticus *peptidases have also been shown to accelerate the ripening process [32,33].

It has been previously reported that there are differences in the proteolytic system of LAB that occupy different environmental niches [12]. Dairy strains such as *Lb. helveticus *CP70, *Lb. bulgaricus *SS1 and *L. lactis *subsp. *cremoris *FT4 have been shown to harbor proteolytic enzymes which release bioactive peptides from milk [34]. This is not fully reflected in our results as we found only two *Lb. helvetic*us DPC4571 genes, lhv_1161 and lhv_1171, that were unique to dairy and multi-niche organisms, both of which are carboxypeptidases from the M20/M25/M40 metallopeptidase family. The role of metallopeptidases in LAB is not fully understood but they could play different roles at the physiological and technological level. These proteins could be involved in bacterial growth by supplying amino acids; for example, PepS has been shown to release phenylalanine and arginine, which are known to stimulate the growth of *S. thermophilus *CNRZ302 in milk. Metallopeptidases may also participate in the development of flavour in food products, either directly, by hydrolysing bitter peptides which are generally rich in hydrophobic amino acids and therefore good substrates for its action, or indirectly through the liberation of aromatic amino acids which are precursors of aroma compounds identified in cheese [35]. A broader BLAST search for validation revealed that lhv_1161 and lhv_1171 had homologues in *Listeria, Staphylococcus *and *Bacillus *species, all of which are known colonisers of dairy environments, making lhv_1161 and lhv_1171 ideal dairy LAB identifiers.

### Restriction/Modification Systems

Restriction/modification (R/M) enzymes digest foreign DNA which has entered the cytoplasm while the host DNA remains undigested. R/M enzymes can be sub-classified into 3 groups; Type I, Type II and Type III. Type I enzymes consist of three subunits, which are responsible for modification (M), restriction (R), and specificity (S) and have been designated Hsd standing for host specificity determinant. Three type I R/M enzymes from *Lb. helveticus *DPC4571 are dairy organism-specific; hsdR (lhv_1031), hsdS1 (lhv_1152) and hsdR (lhv_1978). Also, there is one dairy specific type III R/M enzyme mod (lhv_0028). A broader BLAST search confirmed that these genes only occurred in organisms capable of survival in a dairy environment with homologues in *Pediococcus, Ruminococcus *and *Clostridia *species. These 4 restriction modification genes, lhv_1031, lhv_1152, lhv_1978, lhv_0028 are therefore suitable for inclusion in our barcode as dairy specific genes.

It is not clear as to why these R/M proteins are found only in the dairy organisms and not those found in a gut environment. One possibility may be that higher populations of bacteria are present in the dairy environment they may be more susceptible to phage attacks and therefore require more R/M pathways. The dairy environment usually involves the growth of the starter strains to numbers that are very high when compared to the numbers reached by similar species in other environmental niches and the same starter strains are often used repeatedly over extended periods of time. Use of starter cultures in this manner is known to facilitate the development of populations of bacteriophage specific to the cultures in use. Prior to the development of modern defined strain starters the starter used in milk fermentations would have contained a number of different strains and over a long period of time strains with r/m systems would be expected to predominate as these systems would offer some protection against bacteriophage attack. Even prior to the development of the modern dairy industry and strain selection techniques the use of back-slopping would ensure that only strains from successful fermentations were propagated in future fermentations. Therefore during the long history of fermented milk products there was a strong selective pressure towards phage resistant strains even before the existence of bacteriophage was known.

### Proposed mechanism of niche adaptation

Niche adaptation occurs in a number of ways, namely gene loss or decay, lateral gene transfer or gene up regulation or mutation. In LAB, there is evidence for all of these mechanisms. The high number of pseudogenes in the dairy LAB provides us with striking evidence of gene loss (Table 1). *Lb. helveticus, Lb. delbrueckii *and *S. thermophilus *have 217, 533 and 180 pseudogenes, respectively, whilst the gut bacteria, *Lb. acidophilus, Lb. johnsonii *and *Lb. reuteri *have no pseudogenes and *Lb. gasseri *and *Lb. salivarius *having just 48 and 49, respectively. These pseudogenes are non-functional due to frameshift, nonsense mutation and deletion or truncation. The functional categories into which these pseudogenes fall is interesting; the majority of the pseudogenes appear to be essential gut-living genes, including those involved in carbohydrate and amino acid metabolism and transport and bile salt hydrolysis. In the case of *Lb. delbrueckii*, the remarkably high number of pseudogenes is indicative of ongoing adaptation and genome specialisation. An example of this is the bile salt hydrolase gene of *Lb. helveticus*, which is frameshifted at nucleotide position 417 which introduces a stop codon, rendering the gene inactive.

There is also strong evidence of lateral gene transfer events in the form of fluctuations in the GC content of the genomes. *Lb. delbrueckii *has a higher than average GC content of 49%, mostly due to differences at codon position 3. The evolution at codon position 3 is much faster than position 1 or 2, suggesting that *Lb. delbrueckii *is in an active state of genome evolution[36]. Within the *Lb. delbrueckii *genome, there is still evidence of lateral gene transfer with regions of GC content as high as 52%. The most notable of these regions contains an ABC transporter gene which allows protocooperation with *S. thermophilus*. In *Lb. helveticus*, there is a 100 KB section with a GC content of 42% (5% higher that the rest of the genome). Localised within this region are numerous assumed dairy specific genes including those involved in fatty acid metabolism, restriction endonuclease and amino acid metabolism genes [1]. The region is flanked by IS elements and unique 12-bp direct repeat (tcatctactttc) sequences, which further supports the theory that it has been laterally transferred. *S. thermophilus *has more than 50 regions of anomalous GC content, most of which are associated with genes of relevance to milk adaptation. A region of particular interest is a fragment which is 95% identical to the *metC *gene from *Lb. delbrueckii*. The product of the *metC *gene allows methionine biosynthesis, a rare amino acid in milk. This high level of identity suggests a recent lateral gene transfer event between two distantly related species occupying the same environmental niche [13].

These regions of laterally transferred genes are consistent with recently acquired chromosomal regions or genomic islands that have been described in the multi-niche bacterium *Lb. plantarum *[37], but not in the gut specific bacteria. These genomic islands are thought to increase the ability of *Lb. plantarum *to adapt to multiple environmental niches [38]. Of the other multi-niche bacteria, they have evolved in different ways to be able to adapt to multiple niches. *Lb. sakei *was isolated from meat but can also survive the gut. To this end, it has acquired (most likely through lateral gene transfer) numerous additional metabolic and stress genes allowing it to adapt to a multitude of environmental niches [39]. In specific environmental niches, particularly dairy, plasmids are undoubtedly of significant importance. Plasmids, which are omnipresent in LAB, often encode for genes with technologically important traits and are also seen as major contributors to the metabolic capabilities of a cell. For example, *Lb. salivarius *harbours three plasmids which consist of additional metabolic genes, increasing the overall metabolic capacity and perhaps allowing it to survive in a variety of environmental niches [20].

## Conclusion

The dairy strain *Lb. helveticus *DPC4571 and the gut strain *Lb. acidophilus *NCFM share remarkable genetic relatedness despite coming from such differing niches. We performed an all-against-all BLAST search between *Lb. helveticus *DPC4571 and *Lb. acidophilus *NCFM, which identified 626 genes that differed between the two, potential niche identifier genes. Using a threshold of 1e^-10 ^and greater than 30% identity for homologue detection we searched each of the 626 genes against an eleven genome group. From this analysis 9 genes emerged as being niche specific i.e., genes which were found solely in organisms associated with the gut or genes found solely in organisms associated with the dairy environment. We observed that these 9 genes were involved in characteristics desirable for gut or dairy survival, namely sugar metabolism, the proteolytic and R/M systems and bile-salt hydrolysis. Simultaneously to this unbiased bioinformatic test we examined in depth all genes involved in dairy and gut characteristic traits for niche-specific genes and interestingly we ended up with the same 9 gene "barcode". These 9 "barcode" genes were further validated by performing wider homology searches, using the same homology detection thresholds to ensure that the gut-specific genes were not present in other dairy organisms and vice versa and the 9 gene "barcode" was maintained.

This gene set while limited may provide a useful initial guide to researchers to probe a strains genetic origin. We propose that using the gene-set as a guide; researchers may be able to design primers for their desired "niche" and determine the organism's ability to survive the niche. Undoubtedly this barcode will have to be continuously monitored and further validated as more genomes are sequenced to uphold its accuracy. Additionally there is always the potential for dairy organisms to be introduced to the gut environment through functional food which may lead to them evolving to survive in this environment, for this reason also, we must constantly monitor and update the barcode.

## Methods

### Genome Sequences

Eleven LAB genomes were selected for analysis. Five from a gut environment; *Lb. gasseri *ATCC 33323 [NCBI:CP000413] [5], *Lb. acidophilus *NCFM [NCBI:CP000033] [2]*Lb. johnsonii *NCC533 [NCBI:AE017198] [5], *Lb. salivarius *subsp.*salivarius *UCC118 [NCBI:CP000233] [40] and *Lb. reuteri *F25 [NCBI:CP000705] [41] three from a dairy environment; *Lb. helveticus *DPC4571 [NCBI:CP000517] [1], *Lb. delbrueckii *subsp.*bulgaricus *ATCC 11842 [NCBI:CR954253] [36] and *S. thermophilus *LMG 18311 [NCBI:CP000023] [13] and three multi-niche organisms (i.e. can survive in both a gut or dairy environment); *Lb. brevis *ATCC367 [NCBI:CP000416], *Lb. plantarum *WCFS1 [NCBI:AL935263] [37], *Lb. sakei *subsp.*sakei *23 K [NCBI:CR936503] [39] (see tables 1 and 3 for genome features and niche of the genomes). These genomes were chosen based on a number of criteria; their phylogenetic proximity to *Lb. acidophilus *NCFM and *Lb. helveticus *DPC4571, their availability in the public database and their proven ability to survive a dairy or gut niche.

**Table 3:** Source of isolation and environmental niche of the selected LAB

Species	Isolated From	Environmental Niche
***Lb. helveticus *DPC4571**	Cheese	Dairy
***Lb. acidophilus *NCFM**	Infant faeces	Gut
***Lb. johnsonii *NCC533**	Human faeces	Gut
***Lb. sakei *23 K**	Meat	Multi-niche
***Lb. salivarius *UCC118**	Terminal ileum of human	Gut
***Lb. delbrueckii *subsp.*bulgaricus *ATCC11842**	Yoghurt	Dairy
***Lb. plantarum *WCFS1**	Human saliva	Multi-niche
***S. thermophilus *LMG18311**	Yoghurt	Dairy
***Lb. reuteri F275 ***JCM 1112	Adult Intestine	Gut
***Lb. brevis *ATCC3567**	Silage	Multi-niche
***Lb. gasseri ATCC 33323***	Human Gut	Gut

### Determination of the gene set ("Barcode")

The initial selections were based on an unbiased "all against all" comparison of the *Lb. acidophilus *NCFM and *Lb. helveticus *DPC4571 genomes. A manual comparison of the two genomes was undertaken producing a gene list containing potential "gut" genes (those present in NCFM only) and "dairy" genes (those present in DPC4571 only). The differences in the DPC4571 and *Lb. acidophilus *NCFM gene sets were complied visually using ACT (Artemis Comparison Tool) [42] and from GAMOLA [43] BLASTP outputs. The strain specific gene sets were verified by FASTA [44] searches of the DPC4571 and NCFM sequence data using the Kodon software package (Applied Maths, Inc.). From this we established a preliminary barcode of genes which formed the basis for our search of other genomes.

An additional verification of the barcode was performed by a homology search of each of the potential barcode genes against all fully sequenced Lactic Acid Bacterial genomes (source http://www.ncbi.nlm.nih.gov/sutils/genom_table.cgi).

Simultaneously we identified gene-sets of desirable niche-characteristics and performed biased searches within these groups. For each characteristic known genes where identified from ERGO and the literature and BLAST searches were performed against the 11 genome set. From this we established the same barcode of genes as the unbiased test.

### "Barcode" Validation

For each candidate gene in the 'gut' and 'dairy' gene-set, homologous genes, if present, were identified in the 9 other genomes listed above using the Genomic BLAST [45] web server at NCBI. This server is an expansion of the original BLAST [46] program, which allows you to search for homology within specified genomes. Criteria for homologue detection were a threshold of 1e^-10 ^and greater than 30% identity. Genes which were determined to be suitable for the barcode, based on 'gut' or 'dairy' criteria, were further validated through a BLAST search against a non-redundant database. If a potential gut identifier gene was found in a non-gut organism outside of our initial ten organisms, it was not included in the barcode. The same rule was followed for potential dairy identifier genes.

### Phylogenetic analysis

A phylogenetic supertree was constructed using 47 ribosomal proteins from the 12 species, as well as from *Bacillus subtilis *which was used as an outgroup as previously reported [6]. Proteins were individually aligned using ClustalW [47] and protein trees were built using the PHYLIP [48] package. The best supertree was found using the Most Similar Supertree (dfit) and Maximum Quartet fit (qfit) analysis methods from the Clann package [49].

## Authors' contributions

OOS Primaryauthor, experimental design and contributed to all experiments. JOC reviewed, sugar metabolism work and intellectual contribution to the manuscript. ASV Contributed to experiments. OMcA contributed to experiments and reviewed manuscript. LS contributed to experiments. PK contributed to experiments. MC experimental design and intellectual input. GF Principal investigator and intellectual input RPR Principal investigator and intellectual input. TB Principal investigator and intellectual input. All authors have read and approved the final manuscript.
